# Mr. William Rose

**Published:** 1910-06-04

**Authors:** 


The death is announced of Mr. William Rose, at the age of 62. His brilliant career began at King's College and continued through the membership of the R.C.S. in 1871 to a Fellowship in 1874, till its academic triumph in 1875, when Mr. Rose took a first-class in the honours list in the M.B. and B.S. of London University. The following, among his appointments, illustrate his reputation as a surgeon: Emeritus Professor of King's College, consulting surgeon to King's College Hospital, to the Royal Free Hospital, to St. John's Hospital, Twickenham, and to the G.E. and L.B.S.C. Railway Companies. He had also held the posts of registrar and house surgeon to King's College Hospital, and resident clinical assistant to the Consumption Hospital, Brompton. His publications included the "Manual of Surgery" (with Mr. Carless); the "Surgical Treatment of Trigeminal Neuralgia," and various papers to the medical newspapers.

